# A pharmacy led program to review anti-psychotic prescribing for people with dementia

**DOI:** 10.1186/1471-244X-12-155

**Published:** 2012-09-25

**Authors:** Anne Child, Amy Clarke, Chris Fox, Ian Maidment

**Affiliations:** 1 Avante Care and Support, De Gelsey House, 1 Jubilee Way, Faversham, Kent, ME13 8GD, UK; 2 NHS Medway, 50 Pembroke Court, Chatham Maritime, Chatham, Kent, ME4 4EL, UK; 3 Faculty of Medicine and Health Sciences, University of East Anglia, Norwich Research Park, Norwich, NR4 7TJ, UK; 4 Pharmacy, School of Life and Health Sciences, Aston University, Aston Triangle, Birmingham, B4 7ET, UK

**Keywords:** Anti-psychotics, National Dementia Strategy, Medication Review, Dementia Registers

## Abstract

**Background:**

Anti-psychotics, prescribed to people with dementia, are associated with approximately 1,800 excess annual deaths in the UK. A key public health objective is to limit such prescribing of anti-psychotics.

**Methods:**

This project was conducted within primary care in Medway Primary Care Trust (PCT) in the UK. There were 2 stages for the intervention. First, primary care information systems including the dementia register were searched by a pharmacy technician to identify people with dementia prescribed anti-psychotics. Second, a trained specialist pharmacist conducted targeted clinical medication reviews in people with dementia initiated on anti-psychotics by primary care, identified by the data search.

**Results:**

Data were collected from 59 practices. One hundred and sixty-one (15.3%) of 1051 people on the dementia register were receiving low-dose anti-psychotics. People with dementia living in residential homes were nearly 3.5 times more likely to receive an anti-psychotic [25.5% of care home residents (118/462) vs. 7.3% of people living at home (43/589)] than people living in their own homes (p < 0.0001; Fisher’s exact test). In 26 practices there was no-one on the dementia register receiving low-dose anti-psychotics.

Of the 161 people with dementia prescribed low-dose anti-psychotics, 91 were receiving on-going treatment from local secondary care mental health services or Learning Disability Teams. Of the remaining 70 patients the anti-psychotic was either withdrawn, or the dosage was reduced, in 43 instances (61.4%) following the pharmacy-led medication review.

**Conclusions:**

In total 15.3% of people on the dementia register were receiving a low-dose anti-psychotic. However, such data, including the recent national audit may under-estimate the usage of anti-psychotics in people with dementia. Anti-psychotics were used more commonly within care home settings. The pharmacist-led medication review successfully limited the prescribing of anti-psychotics to people with dementia.

## Background

Dementia has an estimated global prevalence of 35 million individuals [1]. In the UK 700,000 people live with dementia - a figure which will double over the next 30 years, reflecting global predictions [1-3]. Whilst BPSD (behavioural and psychological symptoms of dementia) are very common, both the point prevalence, the proportion having symptoms at any one time point, and the cumulative risk are unclear. A recent study concluded that BPSD affect nearly everyone with dementia [4]. Whereas the Cochrane review simply stated that 50% of people with dementia experience such symptoms [5]. Other studies have estimated the point prevalence between 60% and 80%, and the cumulative risk during the course of the illness at 90% [6].

BPSD are among the most difficult symptoms to treat, a primary source of caregiver stress, strongly associated with carer burden and depression and one of the main drivers for placement breakdown and admission to residential care facilities [7-12]. Behavioural symptoms have traditionally been treated with anti-psychotics, however their usage is implicated in approximately 1,800 annual deaths [2]. Recently the National Dementia Strategy (NDS) targeted a two-thirds reduction in such usage of anti-psychotics [2,13-15]. However, there is limited information on the prevalence of anti-psychotic use in dementia and the NDS estimated that, as a conservative figure, up to 25% of people with dementia are likely to be receiving anti-psychotics [2]. Due to the lack of data, one of the initial recommendations of the NDS was to obtain baseline data on the extent of anti-psychotic usage [2]. Following collection of baseline data a target for the size and speed of the reduction should be set; the NDS also highlighted the potential role of pharmacists in improving medication management and limiting the inappropriate use of anti-psychotics in people with dementia [2,14,16].

## Method

### Setting

This pharmacy-led program was conducted within General Practitioner (GP) surgeries in Medway Primary Care Trust (PCT). Medway PCT covers the Medway towns in Kent with a total population of 256,700 and a 60 plus population of 51,500 [17]. Medway is a relatively deprived area, ranked 132rd most deprived of 325 UK boroughs in the 2010 Index of Deprivation, with pockets of significant deprivation; eight geographical units called ‘Super Output Areas’ (SOAs) were ranked in the 10% most deprived nationally [18].

Pharmaceutical support within Medway PCT is provided by a medication management team containing pharmacists, pharmacy technicians and support staff; the team has a history of a close working relationship with GPs and undertaking clinical projects within individual practices. Following the publication of the NDS one of the key strategic priorities of the PCT was to limit the prescribing of anti-psychotics to people with dementia. This project, which was conducted from January to December 2011, had two aspects. First, in line with the NDS, data were collected on anti-psychotic usage to identify people on the dementia register within Medway PCT prescribed anti-psychotics. Second, a specialist pharmacist intervened in an appropriate cohort of people with dementia commenced on anti-psychotics by primary care identified by the data search.

### Patient identification

Every practice information system, both the electronic and paper format, was searched, by an experienced pharmacy technician to identify people with dementia currently prescribed a low dose anti-psychotic, on acute or repeat prescription. Dementia registers were established within the UK as part of the Quality Outcome Framework (QOF) – an incentive scheme to reward good practice in primary care - to record people with dementia within the practice [19,20]. The registers were established within Medway PCT in 2006/07 and were used to identify people with a confirmed diagnosis of dementia. The individual patient record including the medication history of every person on the dementia register was then searched to identify people prescribed low-dose anti-psychotics. Initially, this was restricted to the 6 most commonly prescribed anti-psychotics based on ePACT (electronic Prescribing Analysis and Cost Tabulation) data. However, to ensure completeness, patient’s notes were hand searched to identify other, less frequently prescribed, anti-psychotics. For the six most commonly prescribed anti-psychotics, low dose was defined *a priori* (olanzapine up to 10 mg daily, risperidone up to 2 mg daily, quetiapine up to 100 mg daily, amisulpiride up to 50 mg daily, sulpiride up to 200 mg daily and haloperidol up to 1.5 mg daily). For the other less frequently prescribed anti-psychotics the standard British National Formulary (BNF) dosing schedules were used to inform the decision on whether the dose was low [21].

Finally, the document storage system, where key documents such as referral letters are stored, was searched to determine where treatment was commenced (primary care, secondary care mental health trust, acute trust or Learning Disability Team). If the anti-psychotic had been initiated by a secondary care organisation, the patient was not included in the medication review, because the anti-psychotic may have been used for under-lying psychosis or a complex mental health problem. The residential status (living at home or in a care home) was also collected.

### Pharmacist-led medication review

After the cohort of people with dementia on long-term anti-psychotics initiated by primary care was identified, the intervention was delivered. Practices were ranked according to the frequency of prescribing to prioritise where the intervention should be delivered. The pharmacist delivering the intervention sent a copy of a report to the GP, which identified the cohort of people with dementia where anti-psychotic withdrawal should be attempted and that the pharmacist was able to give support to the GP in the process by providing advice on a suitable withdrawal scheme, advising on local and national guidance and linking with the care home [14,22-24]. This initial communication was followed up either verbally or in writing.

The pharmacist collaborated with the GP in identifying, where withdrawal was potentially suitable and the level of pharmacy support including whether a pharmacist-led medication review, was required. Medication was withdrawn for clinical, not research reasons. Hence, consent was not required. All participants received usual care and any decision to alter medication was made by the GP based on individual clinical needs, following the pharmacist-led review. Withdrawal was generally considered if the patient was not under secondary care services, was receiving an anti-psychotic for non-acute behavioural problems, and the anti-psychotic had not been reviewed in the previous 12 months.

The clinical medication review (based on modified National Prescribing Centre Level 3 medication review criteria) was delivered by an experienced senior clinical PCT pharmacist, who had a further qualification in dementia with full access to medical and care home notes [25]. The review included an individual withdrawal plan, which took into account the length of time the person had been on the anti-psychotic. In general the dose was gradually reduced usually by 50% every two to four weeks following discussions with the prescriber. During the withdrawal patients continued to be monitored, followed up, and treated by their own physicians according to their individual clinical needs. Care home staff were encouraged to log behaviours using ABC (Antecedents, Behaviour, Consequences) charts and monitor residents for any change in general health [23].

Care staff discussed the project with next-of-kin and whenever possible the person with dementia; if required the pharmacist supported these discussions. Any decisions from these discussions incorporated the views of the person with dementia, next–of-kin and professional carers. The pharmacist also provided support and training to the care home, which was designed to help staff manage BPSD, and was available for further advice and follow-up. The training utilised treatment guidelines for use in primary care to treat BPSD that were developed by a multi-disciplinary team of clinicians from the local mental health trust and local PCTs [23]. The overall concept of the training was based upon a person-centred approach including the need to understand the causes of any behaviour and accurately record any behaviour, non-pharmacological treatments options, and the appropriate pharmacological management of behavioural problems in dementia [22,23].

This project was approved by Medway PCT. Ethics committee approval was not required because the project was a service evaluation and data were collected to evaluate this new service and not for the purposes of research. The decision to publish was made retrospectively and individual patient data was not identifiable to anyone in the team (IM, CF) conducting the secondary analysis of the results for publication.

## Results

**Figure 1:**
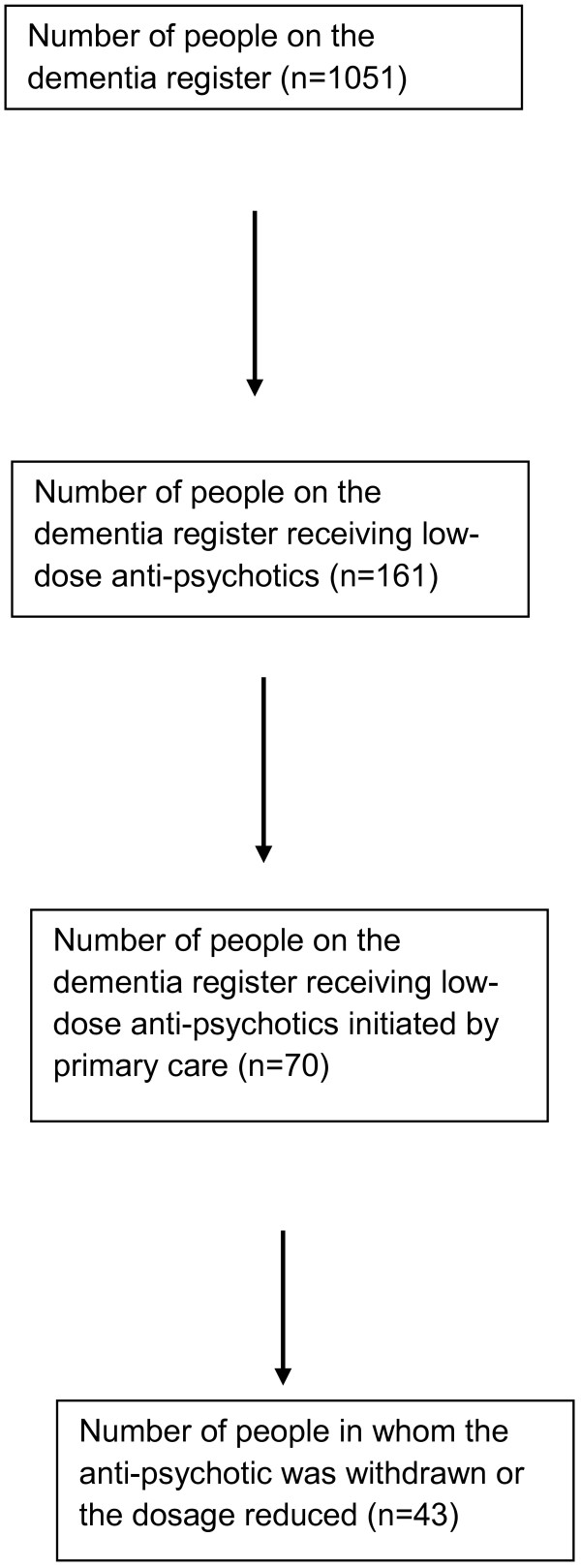
Intervention Flow chart.

See Figure 1 for a diagrammatical representation of the results.

### Anti-psychotic usage

Data were collected from 59 out of 60 (98.3%) practices (or groups of practices) within Medway PCT; one practice declined to take part. There were 1051 people on the dementia register in the 59 practices; of these 462 were in residential care and 589 lived in their own home. In total 161 people on the dementia register were receiving low-dose anti-psychotics; of these 118 were in residential care and 43 lived in their own home. People with dementia living in residential homes were nearly 3.5 times more likely to receive an anti-psychotic; 25.5% (118/462) of people resident in care homes compared with 7.3% (43/589) of people living in their own homes (p < 0.0001; Fisher’s exact test) [26].

On average (mean value) each practice treated 2.77 (range – 0 to 26; +/− SD 4.88) people with dementia with low-dose anti-psychotics. In 26 practices no-one with dementia was receiving low-dose anti-psychotics. Put another way, if one assumes that the dementia register was accurate, approaching half (44.1%) of all practices in Medway PCT were not treating anyone with dementia with a low-dose anti-psychotic. Five practices accounted for over 50% (81/161) of the prescribing; 3 of these 5 practices were significantly larger (16,575, 13,805 and 8,866) than the mean practice in Medway PCT of 4,683 patients (report generated from http://www.epact.ppa.nhs.uk on the 13^th^ April 2012). The remaining two practices were responsible for large care homes.

The most commonly used anti-psychotic was amisulpride (52 out of 161; see Table 1 for full details of anti-psychotics used).

**Table 1:** Details of low-dose anti-psychotics used in people with dementia

**Anti-psychotic**	**N (%)**
Amisulpride	52 (32.3%)
Risperidone	37 (23.0%)
Quetiapine	35 (21.7%)
Olanzapine	14 (8.7%)
Aripiprazole	8 (5.0%)
Haloperidol	7 (4.3%)
Chlorpromazine	2 (1.2%)
Flupentixol	2 (1.2%)
Benperidol	1 (0.6%)
Sulpride	1 (0.6%)
Trifluoperazine	1 (0.6%)
Zuclopenthixol	1 (0.6%)
Total number of anti-psychotics	161 (100%)

### Pharmacist-led medication review

Of the 161 people on the dementia register prescribed low-dose anti-psychotics, 87 were receiving on-going treatment from local secondary care mental health services and 4 from the local Learning Disability Teams. The remaining 70 patients were included in the pharmacy led review. Different surgeries accessed different levels of pharmaceutical support and in total, following the pharmacy-led intervention, anti-psychotics were withdrawn, or the dosage of the anti-psychotic was reduced, in 43 instances (61.4%). The pharmacist directly liaised with 11 practices to withdraw anti-psychotics in 33 people (47.1%). In a further two practices, one which supported a specialist dementia home, six patients identified during data collection were withdrawn from anti-psychotics. In one further practice, which supported a large EMI (elderly mentally ill) home, the pharmacist developed and provided withdrawal schedules for four patients.

## Discussion

Over 15% of people on the dementia register were receiving low-dose anti-psychotics. This compares with the NDS, which estimated a point prevalence of 25% [14] and an observational study, involving a random sample of GP practices across five primary care trusts, which found that 26% of people with dementia were prescribed anti-psychotics [27]. The recent national audit calculated that in 2011, nationally 6.8% and within Medway PCT 10.5% [Personal Communication with Jonathan Hope] of people with dementia received an anti-psychotic [3]. However, only 45.7% of GP practices across England participated in the national audit due to technical issues or insufficient resources for data extraction [3]. Across Medway PCT 17.5% of practices participated in the national audit compared with 98.3% of practices in this project [3].

Both the national audit and this project relied upon the accuracy of the dementia registers. However, in many cases a formal diagnosis of dementia may not be recorded [3]. A typical GP practice would contain between 12 to 20 people with dementia and between three to five people with dementia on low-dose anti-psychotics [14,28]. In this project 26 practices failed to report anyone on the dementia register receiving low-dose anti-psychotics. The dementia register was set-up in 2006/07 in Medway and should have been well-established. However, an Alzheimer’s Society national survey identified significant under diagnosis; in Medway only 43.8% of the expected number of people with dementia received a diagnosis, which reflects the national picture [29]. This confirms findings elsewhere e.g. NHS Dumfries and Galloway estimated that the QOF dementia register only contained 32.9% of the expected dementia population [30]. Therefore, both the national audit and this project may under-estimate the number of people with dementia prescribed anti-psychotics.

To confirm the extent of the usage of anti-psychotics in dementia, an alternative approach to solely relying on the dementia register may be required. One such alternative option would be to initially identify everyone aged over 65 on low-dose anti-psychotics. *A priori* criteria for a diagnosis of dementia including a prescription for a cognitive enhancer, in addition to presence on the dementia register, could then be applied. This population identified by the alternative approach should be compared to that identified by solely relying upon the dementia registers, and if it captures the true magnitude of the issue used, as entry criteria for any future intervention.

Prescribing was concentrated in a relatively small number of practices and a person with dementia in a residential home was nearly 3.5 times more likely to receive a low-dose anti-psychotic than someone with dementia living in their own home. This confirms earlier research, which highlighted that anti-psychotic use for people with dementia in nursing homes was becoming increasingly prevalent [31]. Therefore, to achieve maximum benefit, and in line with the NDS, and the approach adopted in this project, baseline data should be used to target where the intervention is delivered [14].

The most commonly used anti-psychotic was amisulpride, followed by risperidone and quetiapine. These three anti-psychotics were also identified by an expert group of old age consultant psychiatrists as the most appropriate treatments for challenging behaviour in dementia [32]. However, the efficacy of amisulpride for this indication is unproven, and although there may be less harm associated with quetiapine, there is no evidence that quetiapine is effective in people with dementia [22,33-35]. Furthermore, neither amisulpride nor quetiapine are licensed to treat the behavioural and psychological symptoms of dementia. The licensed treatment, risperidone, which is recommended by local and national guidelines, was only used in 23.0% of instances [22,23]. Risperidone was one of the first anti-psychotics highlighted by the Committee of Safety on Medicines to cause excess mortality and local and national guidelines, which highlight that all anti-psychotics carry a similar risk, appear not to have been fully communicated to prescribers [24,32,36].

There are various limitations with the project in addition to issues highlighted regarding the accuracy of the dementia register. The project was solely conducted within a single PCT, and whilst the PCT covered a very mixed area, the results are not necessarily generalisable to other locations. People initiated on anti-psychotics by secondary care were excluded and a joint pharmacist and GP review, linking with secondary care including experts in the non-pharmacological management of BPSD, might have a more significant impact in limiting the inappropriate prescribing of anti-psychotics [31].

In the short-term, the pharmacy-led intervention reduced the prescribing of anti-psychotics. However, the scope was relatively limited, and excluded other psychotropics, which might be used in place of anti-psychotics [37]. Further, patients were not formally followed-up to determine whether the anti-psychotic was stopped, or simply the dose reduced. The symptoms of BPSD fluctuate and continuing input is likely to be required. To demonstrate such a long-term sustainable quality improvement one would require a full-scale research project which was beyond the scope of this evaluation of a clinical service. An initial step would be a pilot randomised controlled trial (RCT) of a specialist pharmacist-led medication review of psychotropics based upon guidelines from the Alzheimer’s Society [22]. Such a pilot should include a health economic assessment. A positive pilot, and subsequent full trial, could inform future service development.

Key Practice Implications

 – Whilst initial data is encouraging further research is required on the role of pharmacy-led interventions to reduce the prescribing of anti-psychotics and other psychotropics for people with dementia.

 – Any intervention should focus on care homes where the majority of anti-psychotic prescribing appears to occur.

 – The accuracy of the dementia register in identifying people with dementia prescribed low-dose anti-psychotics is unclear.

 – Audit data including the recent national audit may under-estimate the usage of anti-psychotics in people with dementia.

## Conclusion

This project identified that within one PCT approximately 15% of people on the dementia register were receiving low-dose anti-psychotics. However, this may be an under-estimate, because the dementia register may not capture everyone with dementia. Anti-psychotics were used much more commonly within care home settings and the most commonly used anti-psychotic was amisulpride. The project also identified that a pharmacist-led review could successfully limit the prescribing of anti-psychotics to people with dementia.

## Competing interests

CF, AnC and IM have provided consultancy services to pharmaceutical companies marketing psychotropics.

Lundbeck supported a series of Dementia Forums across Kent and Medway involving primary care, secondary care and care home staff to raise awareness about Dementia. Lundbeck had no direct involvement in the project.

## Authors’ contributions

AnC conceived the project and delivered the intervention. AmC collected the data and assisted with data analysis. CF provided clinical input and interpreted the data. IM provided clinical supervision, led the data analysis and drafted the manuscript. All authors commented on earlier drafts, and read and approved the final manuscript.

## Pre-publication history

The pre-publication history for this paper can be accessed here:

http://www.biomedcentral.com/1471-244X/12/155/prepub
